# Risk factors for acute renal injury caused by contrast media after percutaneous coronary intervention and coronary angiography: A protocol for systematic review and meta-analysis

**DOI:** 10.1097/MD.0000000000028897

**Published:** 2022-02-18

**Authors:** Junhuan Hou, Guanghua Cao, Junling Liu, Li Cai, Li Zhao, Xue Li

**Affiliations:** Department of Radiology, Army Medical Center of PLA, Chongqing, China.

**Keywords:** contrast-induced acute kidney injury, coronary angiography, meta-analysis., percutaneous coronary intervention, protocol, risk factors

## Abstract

**Background:**

Contrast-induced acute kidney injury (CI-AKI) caused by contrast medium is one of the common complications of percutaneous coronary intervention (PCI)/coronary angiography (CAG). Early identification of the risk factors of CI-AKI in patients with PCI/CAG and help clinical staff to prevent and intervene as soon as possible is very important to improve the clinical outcome of patients. Although domestic and foreign scholars have studied and summarized the risk factors of CI-AKI in PCI/CAG, the conclusions are not the same. Therefore, in this study, meta-analysis was used to summarize the risk factors of CI-AKI in patients with PCI/CAG, and to explore the characteristics of high-risk groups of CI-AKI, to provide reference for early identification and prevention of clinical doctors and nurses.

**Methods:**

We will search related literature of PubMed, Embase, Cochrane Library, Web of Science, China Biology Medicine Database, China National Knowledge Infrastructure, China Science and Technology Journal Database, and Wanfang Database. Eligible studies will be screened based on inclusion criteria, and data extraction, risk of bias assessment, publication bias assessment, subgroup analysis, and quality assessment will be performed. Review Manager version 5.3 software will be used for data analysis. Each process is independently conducted by 2 researchers, and if there is any objection, it will be submitted to the third researcher for resolution.

**Results:**

We will disseminate the findings of this systematic review and meta-analysis via publications in peer-reviewed journals.

**Conclusions:**

The results of this analysis can be used to generate a risk prediction model and provide an intervention strategy for the occurrence of CI-AKI in PCI/CAG.

## 1 Introduction

Contrast-induced acute kidney injury (CI-AKI) is one of the common complications of percutaneous coronary intervention (PCI)/coronary angiography (CAG).^[^1^–^3^]^ At present, the commonly used definition is that serum creatinine increases ≥25% within 48 hours compared with that before radiography, or the absolute value increases ≥0.5 mg/dL (44.2 μmol/L). In the general population receiving PCI/CAG, the incidence of CI-AKI is <3%, but the incidence in high-risk groups is as high as 40%.^[4]^ Patients treated with PCI/CAG had a mortality rate of 7.1% in the backyard of CI-AKI, and the case fatality rate of patients with acute renal failure requiring hemodialysis was 35.7%. Among them, the 2-year survival fatality rate was 81.2%. The incidence of long-term chronic kidney disease and patient mortality increased.^[5]^

At present, the pathogenesis of CI-AKI is not completely clear, and the most important factor may be medulla hypoxia caused by medullary vasoconstriction and direct renal tubular toxicity.^[^6^–^9^]^ Basic renal insufficiency, diabetes mellitus, and excessive dosage of contrast media are the high-risk factors for the occurrence of CI-AKI.^[10]^ Prevention is the key point in the treatment of CI-AKI. However, it is still unable to find accurate early prediction indicators, and can only rely on timely risk assessment to strengthen the prevention of CI-AKI.

At present, there is no unified understanding of the pathogenesis, epidemiological status, diagnosis, and treatment of CI-AKI in PCI/CAG patients,^[^11^–^13^]^ and the research results of risk factors are not consistent.^[^14^–^26^]^ In order to synthesize the results of previous studies, this article will conduct a meta-analysis on the risk factors of CI-AKI in patients with PCI/CAG, in order to identify the risk factors of CI-AKI in patients with PCI/CAG and to provide scientific basis for clinical prevention of CI-AKI in patients with PCI/CAG.

## 2 Methods

### 2.1 Study registration

This protocol has been registered on Open Science Framework grant number DOI 10.17605/OSF.IO/E6Q9A (https://osf.io/e6q9a). This report will be based on the preferred reporting items for systematic review and meta-analysis protocols.^[27]^

### 2.2 Eligibility criteria

Inclusion criteria:

(1) Study design: we will include all observational studies (case-control study, cohort study, prospective study, etc) that analyze the correlation between risk factors and CI-AKI in patients with PCI/CAG.
(2) Participants: we will include adults identified with the CI-AKI in patients with PCI/CAG who are aged 18 years and above.
(3) Diagnosis of CI-AKI: CI-AKI, defined as an increase in serum creatinine after exposure to contrast media. CI-AKI caused by the contrast media; exclusion of renal damage caused by other diseases.
(4) Outcomes: the results of the study involve the specific values of odds ratio and 95% confidence interval (95% CI) of risk factors.

Exclusion criteria:

(1) the full text cannot be obtained normally, or the extracted data is affected.
(2) repeatedly published literature.
(3) review, systematic review, conference and animal experiments, and other literature.

### 2.3 Search strategy

Electronic databases include PubMed, Embase, Cochrane Library, Web of Science, China Biology Medicine Database, China National Knowledge Infrastructure, China Science and Technology Journal Database, and Wanfang Database. The search terms include contrast-induced acute kidney injury, CI-AKI, percutaneous coronary intervention, PCI, coronary angiography, CAG, angiocardiograph, acute kidney injury, AKI, contrast-induced nephropathy, acute renal insufficiency, risk factor, risk assessment, multivariate analysis, and multivariable logistic regression. The search date is from establishment of the database to February 2022. These search terms are summarized in Table 1.

**Table 1:** Search strategy in PubMed database.

Number	Search terms
#1	Contrast-induced acute kidney injury[Title/Abstract]
#2	CI-AKI[Title/Abstract]
#3	Acute kidney injury[MeSH]
#4	Acute Kidney Failure[Title/Abstract]
#5	Acute Kidney Insufficiency[Title/Abstract]
#6	Acute Renal Failure[Title/Abstract]
#7	Acute Renal Injury[Title/Abstract]
#8	Acute Renal Insufficiency[Title/Abstract]
#9	Kidney Failure, Acute[Title/Abstract]
#10	Kidney Insufficiency, Acute[Title/Abstract]
#11	Renal Failure, Acute[Title/Abstract]
#12	Renal Insufficiency, Acute[Title/Abstract]
#13	Acute Kidney Failures[Title/Abstract]
#14	Acute Kidney Injuries[Title/Abstract]
#15	Acute Kidney Insufficiencies[Title/Abstract]
#16	Acute Renal Failures[Title/Abstract]
#17	Acute Renal Injuries[Title/Abstract]
#18	Acute Renal Insufficiencies[Title/Abstract]
#19	Kidney Failures, Acute[Title/Abstract]
#20	Kidney Injuries, Acute[Title/Abstract]
#21	Kidney Injury, Acute[Title/Abstract]
#22	Kidney Insufficiencies, Acute[Title/Abstract]
#23	Renal Failures, Acute[Title/Abstract]
#24	Renal Injuries, Acute[Title/Abstract]
#25	Renal Injury, Acute[Title/Abstract]
#26	Renal Insufficiencies, Acute[Title/Abstract]
#27	Contrast-induced nephropathy[Title/Abstract]
#28	Acute renal insufficiency[Title/Abstract]
#29	or/1-28
#30	Percutaneous Coronary Intervention[MeSH]
#31	Percutaneous Coronary Revascularization[Title/Abstract]
#32	Coronary Intervention, Percutaneous[Title/Abstract]
#33	Coronary Interventions, Percutaneous[Title/Abstract]
#34	Coronary Revascularization, Percutaneous[Title/Abstract]
#35	Coronary Revascularizations, Percutaneous[Title/Abstract]
#36	Intervention, Percutaneous Coronary[Title/Abstract]
#37	Interventions, Percutaneous Coronary[Title/Abstract]
#38	Percutaneous Coronary Interventions[Title/Abstract]
#39	Percutaneous Coronary Revascularizations[Title/Abstract]
#40	Revascularization, Percutaneous Coronary[Title/Abstract]
#41	Revascularizations, Percutaneous Coronary[Title/Abstract]
#42	PCI[Title/Abstract]
#43	Angiocardiography[MeSH]
#44	Coronary angiography[Title/Abstract]
#45	Angiocardiographies[Title/Abstract]
#46	or/30-46
#47	Risk factor[Title/Abstract]
#48	Risk assessment[Title/Abstract]
#49	Multivariate analysis[Title/Abstract]
#50	Multivariable logistic regression[Title/Abstract]
#51	or/47-50
#52	#29 and #46 and #51

### 2.4 Study selection

**Figure 1:**
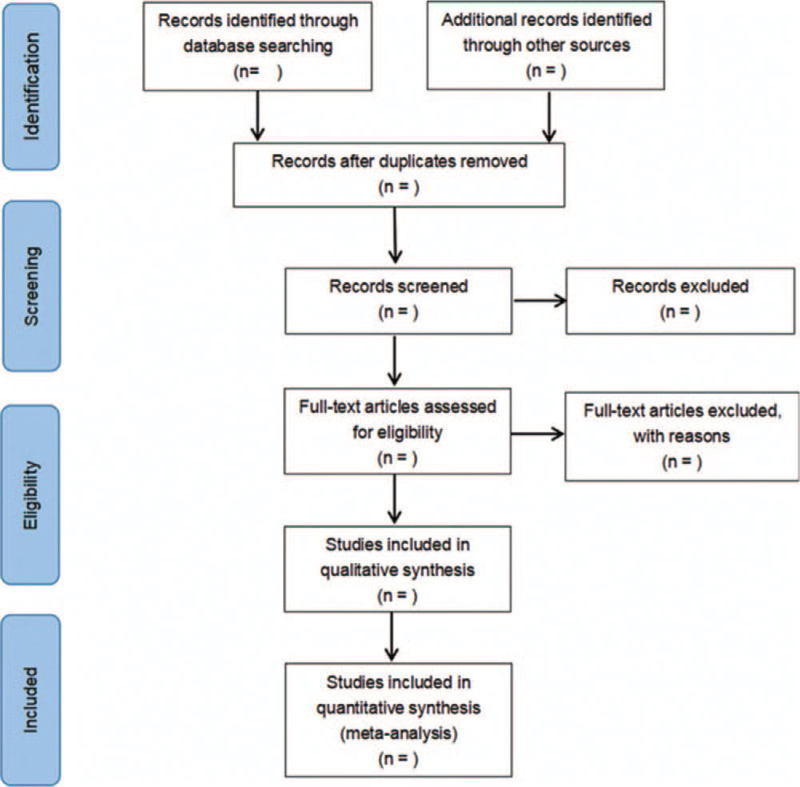
Flow diagram of literature retrieval.

First of all, the original literature was screened by 2 researchers, and the third researcher judged whether to include the literature with conflicting opinions. Secondly, the full text was rescreened according to the detailed entries of the literature inclusion criteria. Finally, the selected literature was analyzed by Meta. The process of the selection is shown in Fig. 1.

### 2.5 Data extraction

The literature data were cross-checked by 2 researchers and then imported into NoteExpress for collation. The main extraction contents include: first author, year of publication, country, research type, study sample size, incidence of CI-AKI, risk factors.

### 2.6 Assessment of risk of bias

Two authors will independently assess the quality of selected articles using the Newcastle-Ottawa scale.^[28]^ Newcastle-Ottawa scale score ≥5 means that the literature quality is better.

### 2.7 Data analysis

The data analysis of this study will be conducted through Review Manager version 5.3 software (Cochrane Collaboration, London, United Kingdom). We will use odds ratio and 95% CI to represent. If there is no finding of statistical heterogeneity, the fixed-effect model is used for data synthesis. If there is significant statistical heterogeneity, we will use the random effect model, and all participants will explore the possible causes from a clinical and methodological perspective and provide a descriptive or subgroup analysis.

### 2.8 Assessment of heterogeneity

The heterogeneity included in the results of the study was analyzed using the chi-squared test (the test level was *α* = 0.1) and combined with *I* ^2^ to quantitatively determine the size of the heterogeneity. When *P* < .1 and/or *I* ^2^ > 50%, the random effect model is used for the combined analysis; otherwise, the fixed effect model is used for the combined analysis.

### 2.9 Subgroup analysis

We make a subgroup analysis according to the type and region of included literature.

### 2.10 Sensitivity analysis

To determine the stability of the outcome measures, each outcome measure was analyzed using sensitivity analysis.

### 2.11 Assessment of reporting biases

We will evaluate the possibility of publication bias using funnel plots and take Egger test of bias as a complement.^[29]^

### 2.12 Confidence in cumulative evidence

We will evaluate the strength of evidence for all outcomes by performing the Grading of Recommendations Assessment, Development and Evaluation working group methodology.^[30]^

### 2.13 Management of missing data

We will try our best to ensure the integrity of the data. If the included data is not complete, we will try every means to contact the corresponding author of the article, including sending emails or making a phone call. If the corresponding author cannot be contacted, we will remove the experiment with incomplete data. After data integrity is assured, intention analysis therapy and sensitivity analysis will be performed.

### 2.14 Ethical review and informed consent of patients

The content of this article does not involve moral approval or ethical review and will be presented in print or at relevant conferences.

## 3 Discussion

In China, about 700,000 people undergo CAG/PCI every year, but 15% to 35% of these patients develop acute kidney injury after surgery.^[^5^,^^31^^]^ In CAG and PCI, the treatment of CI-AKI caused by contrast medium focuses on prevention.^[^32^,^^33^^]^ It mainly includes pre-assessment of the risk of CI-AKI, reasonable and scientific hydration treatment before and after operation, prophylactic use of drugs, rational use of contrast agents, and reducing the use of nephrotoxic drugs during treatment^[^34^–^37^]^ aspects. In clinical practice, for patients undergoing CAG/PCI, we should consider the specific conditions of patients, comprehensively consider various risks, and take a series of treatment and nursing care to prevent the occurrence of CI-AKI. To improve the long-term survival rate and quality of life of patients, patients can avoid damage to renal function while completing CAG/PCI.

As there is no effective treatment of CI-AKI in clinic, hydration is the main treatment.^[38]^ Therefore, understanding the risk factors of CI-AKI is very important for the prevention of CI-AKI. At present, the research on CI-AKI at home and abroad is mainly single-factor and single-center research, while large sample size and multicenter research are few, and the conclusions are different.^[^14^–^26^]^ Therefore, this article conducts a meta-analysis on the risk factors of CI-AKI after PCI/CAG, aiming to provide clinical evidence for the early prevention of CI-AKI.

This study has the following limitations: there are differences in race, number of cases, research tools and regions of this study, and there is a certain heterogeneity after the combination of some risk factors. The purpose of this system review and meta-analysis is to clearly identify the important risk factors of CI-AKI in PCI/CAG in order to provide prevention strategies. In addition, this study will assess new and controversial factors because of their potential as prevention targets.

## Author contributions

**Conceptualization:** Junhuan Hou, Xue Li.

**Data curation:** Guanghua Cao.

**Formal analysis:** Guanghua Cao.

**Funding acquisition:** Xue Li.

**Investigation:** Guanghua Cao.

**Methodology:** Guanghua Cao.

**Project administration:** Xue Li.

**Resources:** Junling Liu.

**Software:** Junling Liu.

**Supervision:** Xue Li.

**Validation:** Junling Liu, Li Cai, Li Zhao.

**Visualization:** Junling Liu, Li Cai, Li Zhao.

**Writing – original draft:** Junhuan Hou, Xue Li.

**Writing – review & editing:** Junhuan Hou, Xue Li.
